# St. Luke’s Hospital

**Published:** 1872-02

**Authors:** John E. Owens


ARTICLE II.—*St. Luke's Hospital.* Oblique fracture of femur at junction of middle and lower thirds; union without shortening; anchylosis from adhesions at seat of fracture. Oblique fracture of femur at junction of middle and upper thirds; union with three-fourths inch shortening. (Under the care of JNO. E. OWENS, M.D.)

J. B., aged 34, of medium size and strength, received a fracture of the right thigh, in consequence of a collision between an engine and passenger coach. He was admitted to the hospital, Dec. 23d, the day he sustained the injury. Upon admission, the leg, on the injured side, was shortened about two and a half inches. A weight and pulley were attached, coaptation splints of sole-leather were applied to the thigh, together with a long external board splint, to prevent the eversion of the foot. The latter splint reached from the brim of the pelvis, a short distance below the foot. (The splint must be sufficiently padded, to prevent excoriations or discomfort.) The treatment commenced with twelve or fourteen pounds, and after a few days this weight was increased to seventeen and a half pounds. At the end of four weeks the dressings were removed; the fracture was firmly united, and both legs gave the same measurement—thirty-three and a half inches.

Any attempt to flex the leg caused considerable pain towards the inner aspect of the thigh, at the location of the fracture. The knee joint itself had a limited motion unattended with pain. Passive motion was employed for three weeks, but proving inadequate to remedy the anchylosis, the patient was ætherized, and, the thigh being well supported, the leg was steadily but slowly flexed to a right angle with the thigh. The rupture of the adhesions was both distinctly heard and felt at the seat of the fracture. The patient was ordered to bed for a few days, and at the end of a week or ten days, the leg was flexed to the normal extent. The patient was discharged from the hospital, March 4th.

March 12th. Extension and flexion are normal—no shortening.

H. W., aged 56, a tall man, February 10th, fell from a scaffold while at work. He was immediately taken to the hospital, where an oblique fracture of left femur was discovered, at the junction of the middle and upper thirds.

A similar dressing to the one used in the above case was employed in this one. The weight which, for the first two weeks, was sixteen and a quarter pounds, was increased to twenty-four and three-fourths pounds. The latter became unbearable, and at the end of forty-eight hours was diminished to twenty and a half pounds. At the end of twenty-two days, the long side splint was dispensed with, and after twenty-five days the dressings were no longer considered necessary. Shortening, three-fourths of an inch.

We are not yet satisfied as to the utility of coaptation splints in fracture of the femur. Precisely the same kind of anchylosis mentioned in the first case, exists in this one. The leg is extended and capable of very little motion. Any attempt to flex the leg causes pulling sensations and pain at the seat of fracture. There was no pain in the knee of the first case, and there is none in this one during flexion. Without a comparison of a number of cases treated with the weight and pulley, with coaptation splints, and with the weight and pulley without these splints, no amount of argument would be profitable.

Anchylosis from adhesions at the site of fracture is a rare occurrence, and yet two exact cases occurring side by side certainly mean more than a coincidence. These are the only cases of the kind that have come to our notice.

In two or three weeks the same treatment will be instituted in this case as was employed in the first one. The fractured bone should be firm, and should be well supported at the time of the forcible flexion, which must be done in a slow and careful manner, under the influence of an anæsthetic, and at two sittings.

We were about to eulogize the treatment of fracture of the femur by means of the weight and pulley, but are somewhat deterred from so doing in consequence of a report in the *London Lancet*, November, 1871, of the treatment of fractured thighs at St. Bartholomew's Hospital, by means of an elastic spring. "An ordinary long splint is provided with a pair of pulley wheels near the upper part, a single one at the opposite extremity, and a row of pegs on the lower half of the outer aspect, for the attachment of the spring and the regulation of the degree of tension. The extremities of the perineal band are attached to the ends of a doubled string, which plays over one wheel of the double pulley, and passes to its attachments over the two upper wheels in the splint. Another string is attached to one end of the spring, passed over the other wheel of the double pulley, and then over the wheel at the lower extremity of the splint, where it is fastened to a stirrup which has been adapted to the patient's ankle. Thus the elastic tension of the spring is brought to bear in an opposite direction upon the two extremities of the limb."

"Three fractured thighs have been treated by its aid at St. Bartholomew's Hospital, and in each instance union has been effected without shortening."

Such results are decidedly brilliant.

